# Aortic pseudoaneurysm on coronary angiography as a cause of complete atrio-ventricular block in COVID-19 patient

**DOI:** 10.1093/ehjci/jeac076

**Published:** 2022-05-02

**Authors:** Maciej T Wybraniec, Radosław Gocoł, Wojciech Wróbel, Małgorzata Cichoń, Katarzyna Mizia-Stec

**Affiliations:** First Department of Cardiology, School of Medicine in Katowice, Medical University of Silesia, 47 Ziołowa St., 40-635, Katowice, Poland; Upper-Silesian Medical Center, Katowice, Poland; Members of the European Reference Network on Heart diseases - ERN GUARD-HEART, Meibergdreef 9, 1105 AZ Amsterdam, Netherlands; Upper-Silesian Medical Center, Katowice, Poland; Department of Cardiac Surgery, School of Medicine in Katowice, Medical University of Silesia, Katowice, Poland; First Department of Cardiology, School of Medicine in Katowice, Medical University of Silesia, 47 Ziołowa St., 40-635, Katowice, Poland; Upper-Silesian Medical Center, Katowice, Poland; First Department of Cardiology, School of Medicine in Katowice, Medical University of Silesia, 47 Ziołowa St., 40-635, Katowice, Poland; Upper-Silesian Medical Center, Katowice, Poland; First Department of Cardiology, School of Medicine in Katowice, Medical University of Silesia, 47 Ziołowa St., 40-635, Katowice, Poland; Upper-Silesian Medical Center, Katowice, Poland; Members of the European Reference Network on Heart diseases - ERN GUARD-HEART, Meibergdreef 9, 1105 AZ Amsterdam, Netherlands

Incidental findings during routine coronary angiography (CAG) should prompt further diagnostic evaluation. A 54-year-old male with COVID-19 was referred from temporary infectious hospital with the diagnosis of unstable complete atrio-ventricular block (AVB). Following the initial transthoracic echocardiography, temporary pacing electrode was inserted to right ventricle, leading to the return of hemodynamic stability. CAG revealed an additional chamber originating from left valsalva sinus directly above calcified aortic valve (AV) (*Panel A*). Further CAG showed non-significant narrowing of proximal left anterior descending artery, presumably caused by compression from the additional structure (*Panel B*). This angiographic image triggered transesophageal echocardiography, which revealed large pseudoaneurysm deriving from left sinus of valsalva with large intraluminal thrombus (*Panels C–D*) and moderate stenosis of bileaflet AV. Contrast-enhanced computed tomography of the chest confirmed the diagnosis of large thrombosed aortic pseudoaneurysm adjacent to left coronary artery and AV (*Panels E–G*). Following cardiac surgery consult, a deferred intervention was advised given acute viral pneumonia. A permanent dual-chamber pacemaker was implanted during index hospitalization and further in-hospital stay was uneventful. Six weeks after initial discharge, the patient underwent successful surgical closure of the aneurysmatic orifice with teflon felt-supported sutures and AV replacement with Hancock 27 mm bioprosthesis. The present case underscores the importance of incidental findings during CAG, which may add additional diagnostic value on top of routine transthoracic echocardiography preceding the procedure and require further multimodality imaging. Also, complete AVB may arise from rapidly enlarging aneurysm of aortic sinus, given the anatomical proximity of conduction pathways.

**Figure:**
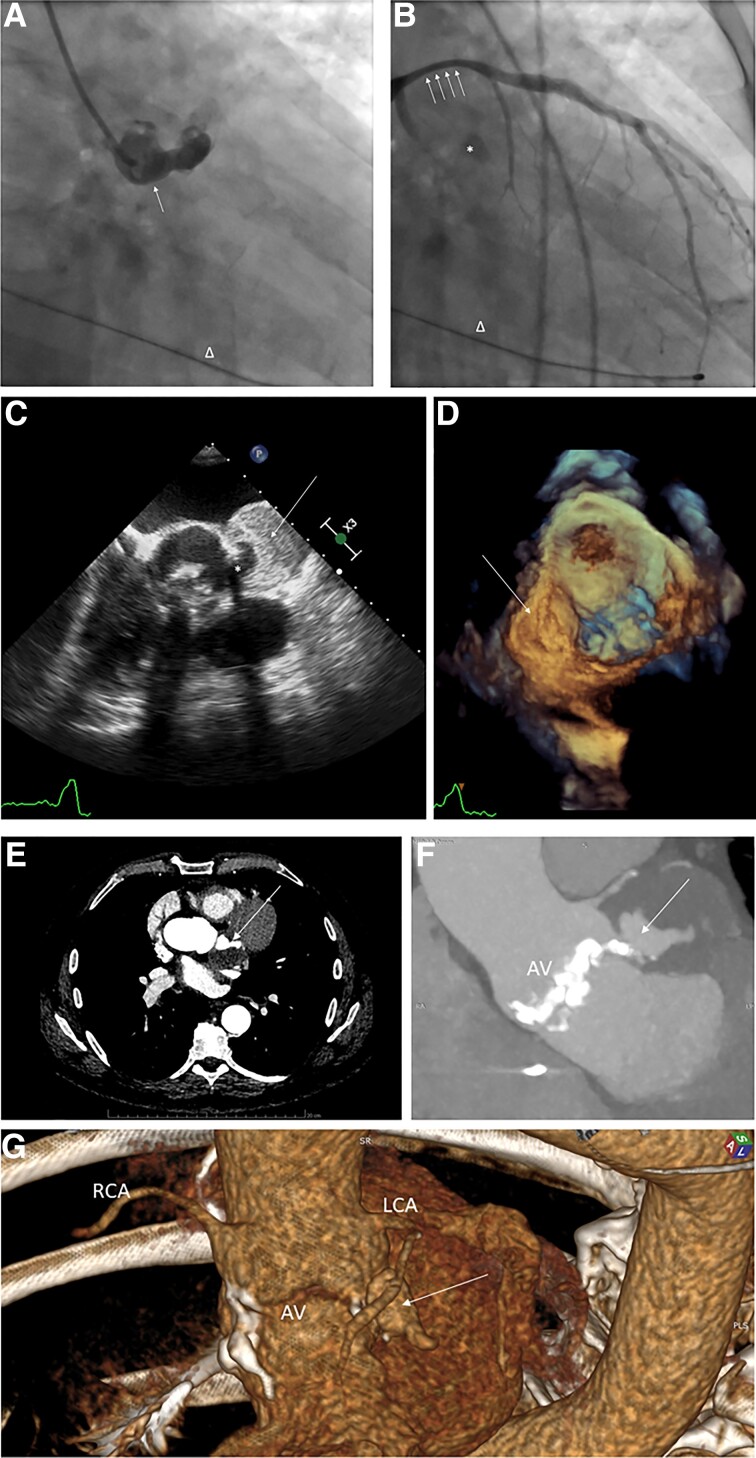


**Conflict of interest**: None declared.

**Funding**: None declared.

